# Supplementary figures and images for: Modified combined short and long axis method versus oblique axis method in adult patients undergoing right internal jugular vein cannulation: A randomized controlled non-inferiority study
Source: PLoS One. 2023 Dec 19;18(12):e0295916. doi: 10.1371/journal.pone.0295916 (PMC10729954; doi:10.1371/journal.pone.0295916)

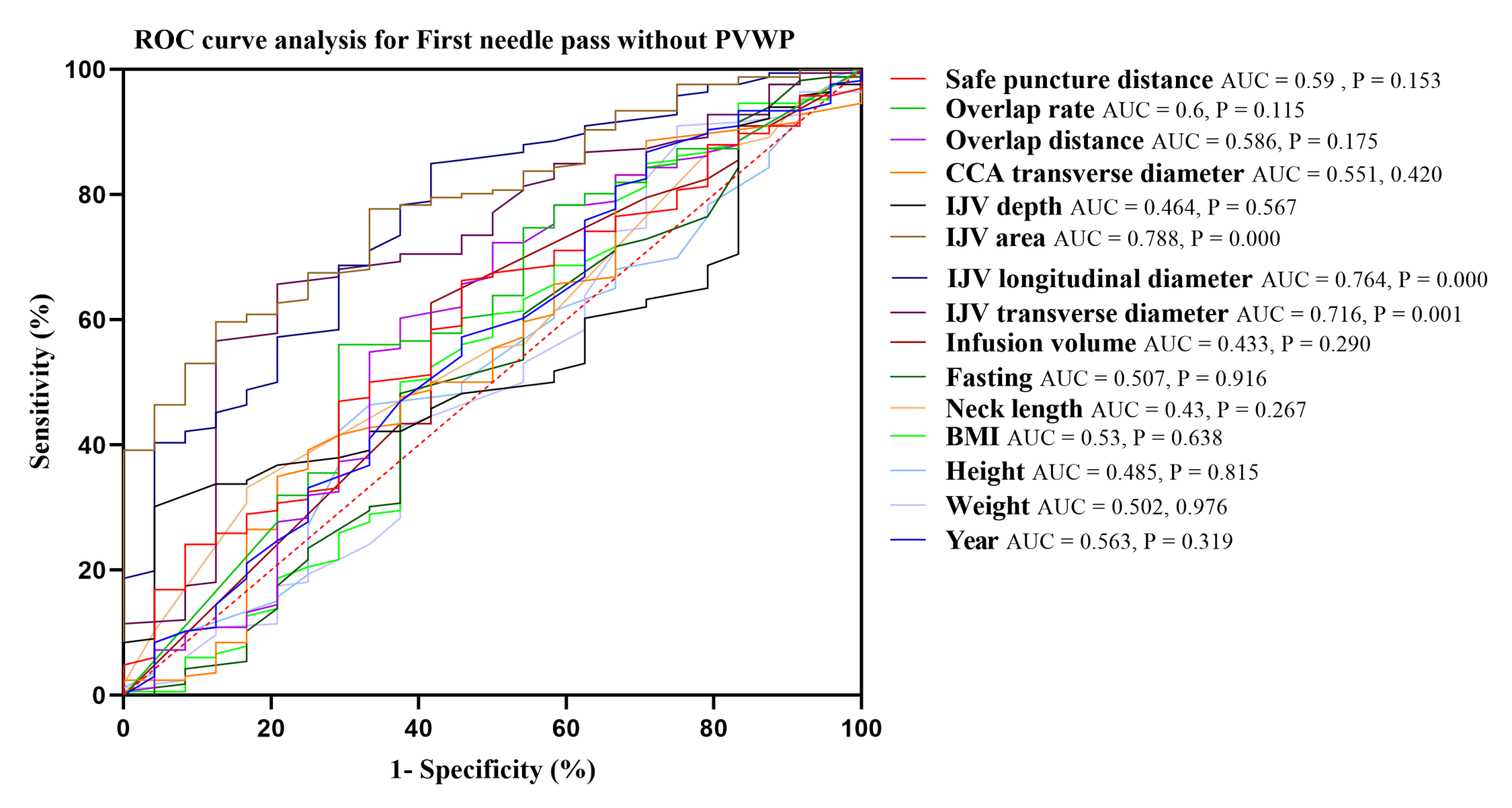

Supplement: S1 Fig — ROC, receiver operating characteristic; PVWP, posterior vessel wall puncture; AUC, Area Under the Curve; CCA, Common carotid artery; IJV, internal jugular vein; BMI, Body Mass Index. (TIF) [file pone.0295916.s001.tif]
