# Supplementary material for: Modified combined short and long axis method versus oblique axis method in adult patients undergoing right internal jugular vein cannulation: A randomized controlled non-inferiority study
Source: PLoS One. 2023 Dec 19;18(12):e0295916. doi: 10.1371/journal.pone.0295916 (PMC10729954; doi:10.1371/journal.pone.0295916)
Supplement: S1 Text — (PDF) [file pone.0295916.s002.pdf]

改良短轴联合长轴法与斜轴法颈内静脉穿刺置管在肥胖患者中的随机对照研究

临床研究方案

版 本 号：1.1

版本日期：2020 年 12 月 3 日

申办者：重庆大学附属肿瘤医院

1. 研究背景

中心静脉穿刺作为临床常规操作，在临床治疗中发挥了巨大作用。其中包括急诊快速输血、输液，有创血流动力学监测，血管活性药物、化疗药物及肠外营养药物输注及血液透析等。颈内静脉（internal jugular vein, IJV）是建立中心静脉通道最常选择的途径之一。随着我国经济的快速发展，现代生活方式和饮食结构的改变，我国成人肥胖人数高达 1.6 亿，肥胖率约 11.9%。传统的解剖标志定位 IJV 穿刺置管在肥胖患者中常导致穿刺置管困难，易出现各种机械并发症，如误穿动脉、血肿、气胸等。超声引导下的颈内静脉置管可明显降低穿刺相关并发症的风险，提高一次性穿刺成功率。

目前临床研究主要集中在短轴法、长轴法、斜轴法以及上述方法的改良方法互做对比，以期寻求最安全高效的超声引导 IJV 穿刺置管方法。大量研究表明斜轴法和改良联合方法显示出了优势。本研究拟通过对比斜轴法和改良短轴联合长轴法，研究斜轴和短轴下肥胖患者超声解剖的差异，并且对比联合方法和斜轴法，在穿刺时间，穿刺成功率及穿刺并发症方面的差异，找到最适合肥胖患者的超声引导 IJV 穿刺方法，并进行技术推广，提升区域医疗技术水平，具有重要临床意义。

2. 研究目的

拟评价改良短轴联合长轴法在右颈内静脉穿刺置管技术的安全性及有效性，对比其和斜轴法在穿刺成功率及穿刺并发症方面的差异，找到最适合肥胖患者右颈内静脉穿刺的方法，并进行技术推广，提升区域医疗技术水平。

3. 研究方案

1）研究设计

这是一项单中心、随机、对照、非劣效，评估者盲的临床研究。研究选择改良短轴联合长轴法作为试验组，斜轴法作为对照组，按照 1:1 均衡分配，计划纳入 190 例受试者。

2） 纳入标准

(1)受试者自愿加入本研究，签署知情同意书，依从性好；

(2)年龄：18-75 周岁（签署知情同意书时）；

(3)就诊我院，择期手术，拟行右侧颈内静脉穿刺置管的患者。

3） 排除标准

(1)凝血功能异常；(2)穿刺部位感染；（3）ASA 分级>III 级；(4) 颈部瘢痕；(5)颈部外伤或手术史患者；(6)1 月内右颈内静脉置管史；(7)存在血液系统疾病者；(8)精神性疾病患者；(9)解剖变异，无右 IJV 者；(10) IJV 斑块血栓形成；(11)超声显像不清晰者。

4）分组及随机

采用电脑产生随机数字表。手术前一天术前访视时，参与麻醉访视的住院医师，根据纳入排除标准，和患者签署临床研究知情同意书。根据区组为 4，随机将入选患者 1：1 分入 2 组：即 M 组：改良短轴法联合长轴法组，O 组：斜轴法组，每组各 95 人。

5）盲法

为增加研究结果及由此推论所得结论的可靠性，在综合衡量研究的可行性基础上，随机数字表封存于非透明信封，由经过专门培训的不参与本实验的专职人员保管。由于操作本身特点，操作者无法实施盲法，本研究对患者及参与数据记录、整理人员采用盲法。

6）试验内容

6.1 药物与试验设备

6.1.1 药物

羟乙基淀粉（万衡），国药准字 H81LK111，规格：500 ml，生产企业：北京费森尤斯卡比医药公司

钠钾镁钙葡萄糖注射液（乐加），国药准字 H180514EN，规格：500 ml，生产企业：江苏恒瑞医药股份有限公司

复方氯化钠注射液，国药准字 H191536LK，规格：500 ml，生产企业：湖北宜昌人福药业有限责任公司

安必洁医用超声耦合剂（安欣超），渝食药监械（准）字 2014 第 2230176

盐酸利多卡因注射液，国药准字 H22020052, 规格：5 ml，生产企业：吉林康乃尔药业有限公司

6.1.2 设备

一次性无菌单腔/双腔中心静脉导管包（艾贝尔），生产企业：广东百合医疗科技股份有限公司

一次性无菌保护套，生产企业：广州雅夫生物科技有限公司

监护仪，生产企业：荷兰皇家飞利浦公司

Mindray M9T 笔记本式彩色通道超声诊断系统，生产企业：深圳迈瑞生物医疗电子股有限公司

6.2 操作方法

6.2.1 麻醉方法

所有患者入手术室导管准备间后常规监测无创血压、心电图、心率、脉搏氧饱和度，开放外周静脉。局部麻醉下行右颈内静脉穿刺置管术。

6.2.2 穿刺及图像采集方案

所有右颈内静脉穿刺置管操作均由同一名熟练掌握两种方法的高年资主治医师完成。使用的是单人技术（由同一位操作者操作超声探头和穿刺针），使用 Mindray M9T 笔记本式彩色超声诊断系统的 12L5-A 高频探头，探头长度为 4.7cm（设置频率为 7.5-10 MHz，深度为 5cm）。患者麻醉后，去枕平卧，头偏向左侧 30°，头低脚高 15°。在准备皮肤消毒前，先进行术前超声检查，辨别图像中 IJV 和 CCA（一般 IJV 相比 CCA 位置更表浅，血管壁薄，直径粗，管腔呈椭圆形，大小可随呼吸变化，探头加压可将其压扁；CCA 位置较深，血管壁厚，直径细，管腔呈圆形，探头加压不能将其压扁。采用彩色多普勒超声成像技术，探头朝向近心端，可以看到红色血流信号区域为 CCA，而蓝色血流信号区域为 IJV），确认 IJV 通畅。在甲状软骨水平观察所有患者短轴平面与斜轴平面 IJV 与 CCA 毗邻关系并采集超声图像并保存图像。

保存图像后，根据分组情况，根据分组情况，行改良短轴法联合长轴法和斜轴法穿刺。首先常规消毒，铺无菌洞巾，将超声探头放进无菌保护套，往保护套内注入 10 ml 无菌超声耦合剂，固定好探头套，其中改良短轴法超声探头提前在探头中心线位置固定不透射线的钡线。两组操作方法如下：

M 组（改良短轴联合长轴法）：将超声探头横向放置在右侧胸锁乳突肌三角（胸锁乳突肌下端锁骨头、胸骨头与锁骨上缘构成的三角）的顶点处（与锁骨平行），探头的 Mark

点朝向患者内侧，可以观察到 IJV 和 CCA 的短轴视图。调节探头左右位置，使探头锁线的声学阴影正好落在 IJV 中点的正上方，局麻后，穿刺针从探头中点平面外进针，在超声屏幕上见一高回声亮点时停止进针，将超声探头顺时针旋转 90°，即可见 IJV 的长轴切面及穿刺针针体，超声引导下进针，当在超声图像中看到针尖刺破血管壁并有暗红色血液回流通畅至针筒，停止进针，停止超声探查，置入导丝，超声短轴再次确认导丝位置正确，置入中心静脉导管，回抽有暗红色血液确认中心静脉导管放置在颈内静脉，固定导管，覆盖无菌透明敷贴。

O 组（斜轴法）：将超声探头横向放置在右侧胸锁乳突肌三角（胸锁乳突肌下端锁骨头、胸骨头与锁骨上缘构成的三角）的顶点处（与锁骨平行），探头的 Mark 点朝向患者内侧，可以观察到 IJV 和 CCA 的短轴视图。随后将探头顺时针旋转 45°，使探头的 Mark 点在近心端朝向患者对侧乳头，即获得 IJV 斜轴平面，局麻后，穿刺针从探头外侧 0.5～1 cm 平面内进针，穿刺过程中实时观察穿刺针进程，当在超声图像中看到针尖刺破血管壁并有暗红色血液回流通畅至针筒，停止进针，停止超声探查，置入导丝，超声短轴再次确认导丝位置正确，置入中心静脉导管，回抽有暗红色血液确认中心静脉导管放置在颈内静脉，固定导管，覆盖无菌透明敷贴。

6.2.3 穿刺并发症预案及处置方案

1） 研究过程中使用的局部麻醉药物及输注的液体所致的过敏反应。我们会严格筛选患者，详细询问药物过敏史及家族史，准备好过敏抢救药物及设备。一旦发生过敏反应，立即停止用药。轻度过敏反应，给予抗过敏药物治疗；发生严重过敏反应，启动严重过敏反应应急预案。

2）中心静脉穿刺相关的风险：

最常见的不良反应有轻度局部出血、针刺部位疼痛；皮肤下肿胀并含血（血肿）等，可通过局部按压，追加局部麻醉药物等措施进行处理；罕见但严重的不良反应有感染，气胸，血气胸，动脉损伤，臂丛神经损伤，甲状腺损伤，乳糜漏，穿刺失败等，可通过严格无菌消毒，选择有经验的操作者，轻柔操作，调整超声设备参数，清晰显示颈内静脉周围重要组织结构。如遇到穿刺困难及失败患者，及时终止操作，该患者认定为穿刺失败，退出本研究，邀请更高年资的医师，更换穿刺部位，完成中心静脉穿刺置管。如怀疑发生相应并发症，医师通过临床体格检查，结合超声及影响检查，及时确诊相应并发症，进行及时的处理和治疗。

7）结局指标

7.1 主要结局指标

①无 IJV 后壁（posterior vessel wall puncture，PVWP）穿透的一次穿刺成功率（穿刺针进入 IJV，但是回抽无血液，回退穿刺针可见回血，可认定为发生了 PVWP）。

7.2 次要结局指标

①刺入 IJV 时间，定义：从进针开始到超声确认导丝在 IJV 内，并且位置正确。②总的穿刺时间，定义：从进针开始到导管固定，并且位置正确。③短轴平面和斜轴平面下 IJV 横径，CCA 与 IJV 重叠率（[IJV 与 CCA 重叠距离/CCA 横径]×100%），安全穿刺距离=IJV 横径-IJV 与 CCA 重叠距离，CCA 与 IJV 相对位置关系。④穿刺次数（每次穿刺针后退并调整方向视为一次穿刺）。⑤总穿刺成功率（穿刺次数>3 次或出现并发症视为失败，换成其他中心静脉完成穿刺置管）。⑥穿刺并发症，如血肿、误穿动脉、颈外静脉损伤、甲状腺损伤、神经损伤、气胸及血胸等的发生情况（结合穿刺操作者、穿刺操作录像及影像学资料来判断，气胸、血胸的判断由经验丰富的影像科医师根据胸片来判断）。

8）统计学分析

8.1 样本估计

我们假定在没有 PVWP 的情况下，MCSL 和 OA-IP 方法的第一针通过率分别为 95%和 90%。非劣效性界限预先设定为组间主要结局差异为-8%。我们估计每组 85 例患者将在 0.025 水平下提供 90%的功率，以比较 MCSL 方法与 OA-IP 方法。考虑到 10%的脱落率，最终，我们在拟在每组中招募 95 名患者。

8.2 数据管理及统计分析

病例报告表的数据采用双人双份录入，基于 Excel 2010 录入数据并建立分析数据库，经核查确认无误后锁定数据库。采用 SPSS 25.0 软件和 Stata 13.0 软件进行数据的统计分析。连续定量数据以平均值（SD）或中位数（四分位距，IQR）表示。通过 Kolmogorov-Smirnov 检验评估数据分布的正态性。Levene 检验用于检验方差的同质性。对于满足正态分布和独立测量的连续变量，根据方差齐性采用独立样本 t 检验或校正 t 检验进行分析。非正态分布的连续变量采用 Mann-Whitney U 检验进行分析。定性变量数据用数字和百分比表示。根据理论频率和样本量，使用 x2 检验或精确 Fisher 检验分析分类数据。使用逻辑回归和线性回归进行多变量分析以控制可能的混杂因素。P 值

<0.05 被认为具有统计学意义。

9）伦理标准

本试验的方案,病例报告表, 知情同意书均应取得重庆市肿瘤研究所伦理委员会的书面批准后，方可进行。

研究者或研究者授权的人员将负责向每名患者、患者的合法代表或公证见证人解释参加试验的收益及风险，并应在患者进入试验前(手术前 2 天内)取得书面的知情同意。所有由受试者或其合法代表以及主持知情同意过程的人员共同签名并签署日期的知情同意书原件应由研究者保存。

10）资料的保存

研究者应当使资料保存完整，有固定地方存放并落锁保管，以备今后查看。按照我国 GCP 原则，对于研究方，资料保存至少 5 年。

11） 研究进度

拟于 2021 年 2 月召开第一次研究组会议，讨论临床试验方案，确定研究任务。临床试验工作时间拟定 12 个月，临床试验资料的统计处理及各撰写报告约 6 个月。

12） 签字

申办单位：重庆大学附属肿瘤医院

负责人：

2020 年 12 月 3 日

严重不良事件报告表（SAE）

临床研究批准文号： 编号：

<table>
<tr><td>报告类型</td><td colspan="4">☐首次报告 ☐随访报告 ☐总结报告</td><td colspan="2">报告时间： 年 月 日</td></tr>
<tr><td>医疗机构及专业名称</td><td colspan="4">重庆大学附属肿瘤医院</td><td>电话</td><td></td></tr>
<tr><td>申报单位名称</td><td colspan="4">重庆大学附属肿瘤医院</td><td>电话</td><td></td></tr>
<tr><td>实验方案</td><td colspan="6"></td></tr>
<tr><td rowspan="2">受试者情况</td><td>姓名：</td><td>性别：</td><td colspan="3">出生年月：</td><td>民族：</td></tr>
<tr><td colspan="6">疾病诊断：</td></tr>
<tr><td>SAE 情况</td><td colspan="6">☐导致住院 ☐延长住院时间 ☐伤残 ☐功能障碍
☐导致先天畸形 ☐危及生命或死亡 ☐其他</td></tr>
<tr><td colspan="2">SAE 发生时间 ： 年 月 日</td><td colspan="5">SAE 反应严重程度： ☐轻度 ☐中度 ☐重度</td></tr>
<tr><td>实验方案采取的措施</td><td colspan="6">☐继续实施 ☐中止实施</td></tr>
<tr><td>SAE 转归</td><td colspan="6">☐症状消失（后遗征：☐有 ☐无） ☐症状持续 ☐死亡（死亡时间： 年 月 日）</td></tr>
<tr><td>SAE 与实验方案的关系</td><td colspan="6">☐肯定有关 ☐可能有关 ☐可能无关 ☐无关 ☐无法判定</td></tr>
<tr><td>SAE 报道情况</td><td colspan="6">国内： ☐有 ☐无 ☐不详 国外：☐有 ☐无 ☐不详</td></tr>
<tr><td colspan="7">SAE 发生及处理的详细情况：</td></tr>
</table>
