# Supplementary material for: Modified combined short and long axis method versus oblique axis method in adult patients undergoing right internal jugular vein cannulation: A randomized controlled non-inferiority study
Source: PLoS One. 2023 Dec 19;18(12):e0295916. doi: 10.1371/journal.pone.0295916 (PMC10729954; doi:10.1371/journal.pone.0295916)
Supplement: S2 Text — (PDF) [file pone.0295916.s003.pdf]

Randomized controlled study of modified combined short and long axis method and oblique axis internal jugular vein puncture catheterization in obese patients

Clinical Study Protocol

Version number: 1.1

Version date: December 3, 2020

Sponsor: Chongqing University Cancer Hospital

1. Background of the research

As a routine clinical operation, central venipunction has played a great role in clinical treatment. It includes emergency rapid blood transfusion, infusion, invasive hemodynamic monitoring, infusion of vasoactive drugs, chemotherapy drugs, parenteral nutrition drugs and hemodialysis. The internal jugular vein (IJV) is one of the most common options for establishing central venous access. With the rapid development of economy in our country and the change of modern lifestyle and dietary structure, the adult obesity in our country is as high as 160 million, the obesity rate about 11.9%. The traditional anatomic marker location IJV puncture catheterization often leads to the difficulty of puncture catheterization in obese patients, and is prone to various mechanical complications, such as misplaced artery puncture, hematoma, pneumothorax, etc. Ultrasound-guided internal jugular vein catheterization can significantly reduce the risk of puncture-related complications and improve the success rate of one-time puncture.

At present, clinical studies mainly focus on the comparison of short-axis method, long-axis method, oblique axis method and the improved methods of the above methods, in order to find the safest and most efficient ultrasound-guided IJV puncture catheter method. A large number of studies have shown that the oblique axis method and the improved combined method show advantages.By comparing oblique axis method and modified combined short and long axis method,this study intends to study the differences in ultrasonic anatomy of obese patients under oblique axis and short-axis method, and compare the differences in puncture time, puncture success rate and puncture complications between the combined method and the oblique axis method, so as to find the ultrasount-guided IJV puncture method that is most suitable for obese patients, and promote the technology to improve the regional medical technology level. It is of great clinical significance.

2. Objective of the study

To evaluate the safety and effectiveness of modified combined short and long axis technique in right internal jugular vein puncture, compare the difference between it and oblique axis method in puncture success rate and puncture complications, find the most suitable method for right internal jugular vein puncture in obese patients, and promote the technology to improve the regional medical technology level.

3. Research Scheme

1) Research design

This is a single-center, randomized, controlled, non-inferiority, evaluator blind clinical study. The modified combined short and long axis method will be selected as the experimental group and the inclined axis method as the control group, and 190 subjects will be planned to be included in the study according to 1:1 balanced allocation.

2) Inclusion criteria

(1) The subjects voluntarily join the study, sign the informed consent, and the compliance is good;

(2) Age: 18-75 years old (when the informed consent is signed);

(3) Patients who be admitted to our hospital for elective surgery and plan to undergo right internal jugular vein puncture catheterization.

3) Exclusion criteria

(1) Abnormal coagulation function; (2) infection of the puncture site; (3) ASA grade >III; (4) neck scar; (5) patients with a history of neck trauma or surgery; (6) History of right internal jugular vein catheterization within 1 month; (7) Patients with blood system diseases; (8) patients with mental diseases; (9) anatomic variation, no right IJV; (10) IJV plaque thrombosis; (11) the ultrasound imaging was not clear.

4) Grouping and randomization

Generating a random number table using a computer. On the day before surgery, during the preoperative visit, the attending anesthesiologist involves in the anesthesia assessment, will base on inclusion and exclusion criteria, signe the informed consent for the clinical study with the patients. According to a block size of 4, the enrolled patients will be randomized in a 1:1 ratio into two groups: the M group, consisting of 95 patients, receiving the modified combined short and long axis method , and the O group, consisting of 95 patients, receiving the oblique-axis method.

5) Blind method

In order to enhance the reliability of the results of this study and the conclusions derived

therefrom, on the basis of a comprehensive evaluation of the feasibility of the study, the random number table is sealed in a non-transparent envelope and will be kept by specially trained full-time personnel who are not involved in this experiment. Due to the characteristics of the operation itself, the operator could not implement the blind method. In this study, the patients and the personnel involve in data recording and collating will be blinded.

6) Trial content

6.1 Drug and experimental equipment

6.1.1 Drugs

Hydroxyethyl starch (Wanheng), SinopMA Approval number H81LK111, Specifications: 500 ml, Manufacturer: Beijing Fresenius Kabi Pharmaceutical Co., LTD

Sodium Potassium Magnesium Calcium Glucose Injection (LEjia), SINopMA approval number H180514EN, specifications: 500 ml, Manufacturer: Jiangsu Hengrui Pharmaceutical Co., LTD

Compound Sodium Chloride injection, SINopOL H191536LK, specification: 500 ml, Manufacturer: Hubei Yichang Renfu Pharmaceutical Co., LTD

Anbijie Medical ultrasonic coupler (Anxinchao), Chongqing Food and Drug Administration (standard) word 2014 No. 2230176

Lidocaine hydrochloride injection, Sinopharm approval number H22020052, specification: 5 ml, Manufacturer: Jilin Kangnaier Pharmaceutical Co., LTD

6.1.2 Equipment

Disposable sterile single cavity/double cavity central venous catheter kit (Abel), manufacturer: Guangdong Baihe Medical Technology Co., LTD

Disposable aseptic protective sleeve, manufacturer: Guangzhou YAFu Biotechnology Co., LTD

Monitor, manufacturer: Royal Philips, Netherlands

Mindray M9T notebook color channel ultrasonic diagnostic system, manufacturer: Shenzhen Mindray Biomedical Electronics Co., LTD

6.2 Operation Method

6.2.1 Method of anesthesia

Noninvasive blood pressure, electrocardiogram, heart rate and pulse oxygen saturation will be routinely monitored after all patients enter the catheter preparation room in the operating room. Peripheral veins will be opened. Right internal jugular vein puncture catheterization will be performed under local anesthesia.

6.2.2 Puncture and image acquisition protocol

All right internal jugular vein puncture catheterization procedures will be performed by the same senior attending physician who is proficient in both methods. A one-man technique will be used (the same operator operated the ultrasound probe and puncture needle) using the 12L5-A high frequency probe of the Mindray M9T notebook color ultrasound diagnostic system with a probe length of 4.7cm (set frequency 7.5-10 MHz and depth of 5cm). After the patient be anesthetized, the pillow will be removed and the patient lay flat with the head tilted 30 degree to the left and the head low and the foot high at 15 degree. Before preparing for skin disinfection, preoperative ultrasound examination will be performed to identify IJV and CCA in the images (generally IJV is shallower than CCA, the vessel wall is thin, the diameter is thick, the lumen is oval, the size can change with breathing, and the probe can be pressed flat; CCA position is deep, the blood vessel wall is thick, the diameter is thin, the lumen is round, and the probe pressure can not squish it. Color Doppler ultrasound imaging technology will be used, and the probe will be oriented towards the proximal end. It could be seen that the red blood flow signal area is CCA, while the blue blood flow signal area is IJV), patency of the internal jugular vein. The adjacent relationship between IJV and CCA in the short axis plane and oblique axis plane of all patients will be observed at the thyroid cartilage level, and ultrasound images will be collected and saved.

After the images be saved, the modified combined short and long axis method and oblique axis method will be performed according to the group conditions. First, routine disinfection will be performed, sterile tissue will be laid, the ultrasonic probe will be put into the sterile protective cover, and 10 ml of sterile ultrasonic coupling agent will be injected into the protective cover, and the probe cover will be fixed. In the modified combined short and

long axis method, ultrasonic probe will be fixed the radiopaque barium line at the center line of the probe in advance. The two groups of operation methods are as follows:

Group M (modified short-axis combined long-axis method) : The ultrasound probe will be placed transversally at the apex of the right sternocleidomastoid triangle (the triangle formed by the lower end of the sternocleidomastoid and the pectoral bone and the superior margin of the clavicle) (parallel to the clavicle), with the probe Mark With the point facing inwards of the patient, a short-axis view of the IJV and CCA can be observed. Adjust the left and right positions of the probe so that the acoustic shadow of the probe barium line falls just above the IJV midpoint. After local anesthesia, the puncture needle enters the needle from outside the probe midpoint plane, and stops entering the needle when a high-echo bright spot is seen on the ultrasound screen. Rotate the ultrasound probe 90 degree clockwise, that is, the IJV long-axis section and the puncture needle body can be seen. When the needle tip is seen in the ultrasonic image punctured the blood vessel wall and there is dark red blood flow back to the syringe, stop the needle, stop the ultrasonic exploration, insert the guide wire, ultrasonic short axis to confirm the correct position of the guide wire, insert the central venous catheter, draw back dark red blood to confirm the central venous catheter is placed in the internal jugular vein, fix the catheter, cover the sterile transparent patch.

Group O (oblique axis method) : The ultrasound probe will be placed transversally at the apex of the right sternocleidomastoid triangle (the triangle formed by the lower end of the sternocleidomastoid, the thoracic bone and the superior margin of the clavicle) (parallel to the clavicle), and the probe Mark point will be toward the patient's inner side, and the short axis view of IJV and CCA could be observed. Then the probe was rotated 45 degree clockwise so that the Mark point of the probe was facing the patient's contralateral nipple at the proximal end, that is, the IJV oblique axis plane will be obtained. After local anesthesia, the puncture needle was inserted into the plane 0.5 ~ 1cm from the outside of the probe, and the process of the puncture needle will be observed in real time during the puncture process. When it is seen in the ultrasound image that the needle tip punctured the blood vessel wall and dark red blood returned to the syringe smoothly, the needle insertion will be stopped, and then the guide wire will be placed, the ultrasound short axis again confirmed the correct position of the guide wire, the central venous catheter was placed, the dark red blood be drawn back to confirm the

central venous catheter be placed in the internal jugular vein, the catheter will be fixed, and the sterile transparent paste will be covered.

6.2.3 Preplan and treatment plan for complications of puncture

1) Allergic reactions cause by local anesthesia drugs and fluids used during the study.

We will strictly screen patients, ask about drug allergy history and family history in detail, and prepare allergy rescue drugs and equipment. In the event of an allergic reaction, stop the medication immediately. For mild allergic reactions, give anti-allergy medication; In case of severe anaphylaxis, initiate the severe anaphylaxis emergency plan.

2) Risks associated with central venipuncture:

The most common adverse reactions are mild local bleeding and pain at the acupuncture site. Swelling under the skin and containing blood (hematoma), etc., can be treated by local compression, adding local anesthetic drugs and other measures; Rare but serious adverse reactions include infection, pneumothorax, hemophothorax, arterial injury, brachial plexus injury, thyroid injury, chylorrhea, puncture failure, etc. Strict aseptic disinfection can be performed by selecting experienced operators, gentle operation, adjusting ultrasound equipment parameters, and clearly displaying important tissue structures around the internal jugular vein. If the patient encounter puncture difficulties or failure, the operation should be terminated in time, and the patient will be identified as puncture failure and withdrawn from the study. Doctors with higher experience will be invited to change the puncture site. If relevant complications are suspected, the physician can confirm the relevant complications in time through clinical physical examination, combined with ultrasound and impact examination, and conduct timely treatment and treatment.

7) Outcome indicators

7.1 primary outcome indicator

① Successful first needle pass without posterior vessel wall puncture (PVWP). Insertion of the puncture needle into the IJV with no blood aspirated during aspiration, but visible blood return upon withdrawing the needle, which can be regarded as PVWP.

7.2 Secondary outcome indicator

① IJV insertion time, define: from the beginning of needle insertion to ultrasound confirmation that the guide wire be in the IJV and in the correct position. ② Total puncture time, defined: from the beginning of needle insertion to catheter fixation, and the position is correct. ③ IJV transverse diameter, CCA and IJV overlapping rate ([IJV and CCA overlapping distance /CCA transverse diameter] x 100%), safe puncture distance =IJV transverse diameter - IJV and CCA overlapping distance, CCA and IJV relative position relationship. ④ The number of punctures (each time the puncture needle recedes and adjusts the direction is regarded as a puncture). ⑤ The total success rate of puncture (puncture times >3 or complications are regarded as failure, and other central veins are replaced to complete puncture catheterization). ⑥ Puncture complications, such as hematoma, mispuncture artery, external jugular vein injury, thyroid injury, nerve injury, pneumothorax and hemothorax, etc. (combined with the puncture operator, puncture operation video and imaging data to determine the judgment of pneumothorax and hemothorax by experienced imaging doctors according to the chest film to judge).

8) Statistical analysis

8.1 Sample estimation

We assume a first pass without PVWP rate of 95% and 90% for the MCSL and OA-IP methods, respectively. The non-inferiority bound is pre-set as a primary outcome difference between groups of -8%. We estimate that 85 patients in each group would provide 90% of the power at the 0. 025 level to compare the MCSL approach with the OA-IP approach. Considering the 10% shedding rate, ultimately, we intend to enroll 95 patients in each group.

8.2 Data management and Statistical Analysis

The data of case report form was recorded by two persons in two copies. The data was entered in Excel 2010 and an analysis database was established. The database was locked after verification. SPSS 25.0 software and Stata 13.0 software were used for statistical analysis of data. Continuous quantitative data were expressed as mean (SD) or median (interquartile

range, IQR). The normality of the data distribution is assessed by the Kolmogorov-Smirnov test. The Levene test is used to test the homogeneity of the variance. For continuous variables that satisfy the normal distribution and independent measurements, an independent sample t test or a corrected t test is used for analysis based on the homogeneity of variance. Continuous variables with non-normal distribution were analyzed by Mann-Whitney U test. Qualitative variable data were expressed as numbers and percentages. Categorical data were analyzed using the χ^2 test or the exact Fisher test based on theoretical frequency and sample size. Multivariate analysis was performed using logistic regression and linear regression to control for possible confounding factors. P value < 0.05 is considered statistically significant.

9) ethical standards

The protocol, case report form and informed consent form of this study should be approved in writing by the Ethics Committee of Chongqing Cancer Institute before proceeding.

The investigator or investigator authorized personnel shall be responsible for explaining the benefits and risks of participating in the trial to each patient, the patient's legal representative or notarized witness, and shall obtain the patient's written informed consent before entering the trial (within 2 days prior to the operation). All original informed consent forms co-signed and dated by the subject or their legal representative and the person conducting the informed consent process shall be kept by the Investigator.

10) Preservation of the data

Researchers should keep the data intact, have a fixed place to store it and lock it up for future review. According to China's GCP principle, for the research side, the data is stored for at least 5 years.

11) Research progress

The first research group meeting is planned to be held in February 2021 to discuss the clinical trial protocol and determine the research task. The working time of clinical trial is planned to be 12 months, and the statistical processing of clinical trial data and the writing of each report will take about 6 months.

12) Signature

Sponsor Institution: Cancer Hospital Affiliated to Chongqing University

Person in charge:

December 3, 2020

Serious Adverse Event Reporting Form (SAE)

Clinical Study Approval number:: ID:

<table>
<tr><td>Report type</td><td colspan="3">☐Initial report ☐Follow-up report ☐Final report</td><td colspan="2">Report time yr mo day</td></tr>
<tr><td>Medical institution name</td><td colspan="3">Chongqing University Cancer Hospital</td><td>Tel:</td><td></td></tr>
<tr><td>Applicant organization name</td><td colspan="3">Chongqing University Cancer Hospital</td><td>Tel:</td><td></td></tr>
<tr><td>Study protocol</td><td colspan="5"></td></tr>
<tr><td rowspan="2">Participant Status</td><td>Name:</td><td>Gender:</td><td colspan="2">Date of birth:</td><td>Race:</td></tr>
<tr><td colspan="5">Disease Diagnosis:</td></tr>
<tr><td>SAE Status</td><td colspan="5">☐Hospitalization☐ Delayed Hospitalization Time☐ Disability ☐ Functional Impairment
☐ Congenital Abnormality ☐ Critical Life-Threatening or Death ☐Other</td></tr>
<tr><td colspan="2">SAE Occurrence Time: yr mo day</td><td colspan="4">SAE Reaction Severity: ☐ Mild ☐Moderate ☐Severe</td></tr>
<tr><td>Measures Taken in the trail Protocol</td><td colspan="5">☐ Continue to carry out research ☐ Discontinue the study</td></tr>
<tr><td>SAE Outcome</td><td colspan="5">☐Cure (Complications: ☐Yes ☐No) ☐ Symptoms Persistent ☐Death(Date of Death: yr mo day)</td></tr>
<tr><td>Relationship between SAE and study</td><td colspan="5">☐Definitely Related ☐ Possibly Related☐ Possibly Unrelated☐ Unrelated☐ Cannot Determine</td></tr>
<tr><td>SAE Reporting Status</td><td colspan="5">Domestic: ☐Yes ☐No ☐ unknown Overseas: ☐Yes ☐No ☐ unknown</td></tr>
<tr><td colspan="6">Detailed Information on SAE Occurrence and Handling:</td></tr>
</table>
