# Supplementary material for: Modified combined short and long axis method versus oblique axis method in adult patients undergoing right internal jugular vein cannulation: A randomized controlled non-inferiority study
Source: PLoS One. 2023 Dec 19;18(12):e0295916. doi: 10.1371/journal.pone.0295916 (PMC10729954; doi:10.1371/journal.pone.0295916)
Supplement: S1 Table — (DOCX) [file pone.0295916.s004.docx]

CONSORT Statement 2010 - Checklist for Non-inferiority and Equivalence Trials


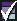


**Items to include when reporting a non-inferiority or equivalence randomized trial**

|  |  | Standard Reporting Item | Non-inferiority and Equivalence Design Reporting Item | Page Number |
| --- | --- | --- | --- | --- |
| **Title and Abstract** |  |  |  |  |
| Title | [#1a](https://www.goodreports.org/reporting-checklists/consort/info/#1a) | Identification as a randomized trial in the title. | ldentification as a non-inferiority/equivalence randomized trial in the title | 1/1 |
| Abstract | [#1b](https://www.goodreports.org/reporting-checklists/consort/info/#1b) | Structured summary of trial design, methods, results, and conclusions |  | 2 |
| **Introduction** |  |  |  |  |
| Background and objectives | [#2a](https://www.goodreports.org/reporting-checklists/consort/info/#2a) | Scientific background and explanation of rationale | Rationale for using a non-inferiority/equivalence design | 3,4/3,4 |
| Background and objectives | [#2b](https://www.goodreports.org/reporting-checklists/consort/info/#2b) | Specific objectives or hypothesis | Hypotheses concerning non-inferiority/equivalence, specifying the non-inferiority/equivalence margin with the rationale for its choice | 4/4 |
| **Methods** |  |  |  |  |
| Trial design | [#3a](https://www.goodreports.org/reporting-checklists/consort/info/#3a) | Description of trial design (such as parallel, factorial) including allocation ratio. |  | 4, 5 |
| Trial design | [#3b](https://www.goodreports.org/reporting-checklists/consort/info/#3b) | Important changes to methods after trial commencement (such as eligibility criteria), with reasons |  | N/A |
| Participants | [#4a](https://www.goodreports.org/reporting-checklists/consort/info/#4a) | Eligibility criteria for participants | Whether participants in the non-inferiority/equivalence trial are similar to those in any trial(s) that established efficacy of the reference treatment | 4, 5/4,5 |
| Participants | [#4b](https://www.goodreports.org/reporting-checklists/consort/info/#4b) | Settings and locations where the data were collected |  | 4 |
| Interventions | [#5](https://www.goodreports.org/reporting-checklists/consort/info/#5) | The experimental and control interventions for each group with sufficient details to allow replication, including how and when they were actually administered | Whether the reference treatment in the non-inferiority/equivalence trial is identical (or very similar) to that in any trial(s) that established efficacy | 5-8/3,4 |
| Outcomes | [#6a](https://www.goodreports.org/reporting-checklists/consort/info/#6a) | Completely defined prespecified primary and secondary outcome measures, including how and when they were assessed | Specify the non-inferiority/equivalence outcome(s) and whether hypotheses for main and secondary outcome(s) are non-inferiority or superiority. Whether the outcomes in the non-inferiority/equivalence trial are identical (or very similar) to those in any trial(s) that established efficacy of the reference treatment | 8, 9/4,9 |
| Outcomes | [#6b](https://www.goodreports.org/reporting-checklists/consort/info/#6b) | Any changes to trial outcomes after the trial commenced, with reasons |  | N/A |
| Sample size | [#7a](https://www.goodreports.org/reporting-checklists/consort/info/#7a) | How sample size was determined. | Whether the sample size was calculated using a non-inferiority/equivalence criterion and, if so, what the non-inferiority/equivalence margin was | 9/9 |
| Sample size | [#7b](https://www.goodreports.org/reporting-checklists/consort/info/#7b) | When applicable, explanation of any interim analyses and stopping guidelines | To which outcome(s) they apply and whether related to a non-inferiority/equivalence hypothesis | N/A/4,9 |
| Randomization - Sequence generation | [#8a](https://www.goodreports.org/reporting-checklists/consort/info/#8a) | Method used to generate the random allocation sequence. |  | 5,6 |
| Randomization - Sequence generation | [#8b](https://www.goodreports.org/reporting-checklists/consort/info/#8b) | Type of randomization; details of any restriction (such as blocking and block size) |  | 5 |
| Randomization - Allocation concealment mechanism | [#9](https://www.goodreports.org/reporting-checklists/consort/info/#9) | Mechanism used to implement the random allocation sequence (such as sequentially numbered containers), describing any steps taken to conceal the sequence until interventions were assigned |  | 5,6 |
| Randomization - Implementation | [#10](https://www.goodreports.org/reporting-checklists/consort/info/#10) | Who generated the allocation sequence, who enrolled participants, and who assigned participants to interventions |  | 5,6 |
| Blinding | [#11a](https://www.goodreports.org/reporting-checklists/consort/info/#11a) | If done, who was blinded after assignment to interventions (for example, participants, care providers, those assessing outcomes) and how. |  | 5,6 |
| Blinding | [#11b](https://www.goodreports.org/reporting-checklists/consort/info/#11b) | If relevant, description of the similarity of interventions |  | 6,7 |
| Statistical methods | [#12a](https://www.goodreports.org/reporting-checklists/consort/info/#12a) | Statistical methods used to compare groups for primary and secondary outcomes | Whether a 1- or 2-sided confidence interval approach was used | 9,10/10 |
| Statistical methods | [#12b](https://www.goodreports.org/reporting-checklists/consort/info/#12b) | Methods for additional analyses, such as subgroup analyses and adjusted analyses |  | N/A |
| **Results** |  |  |  |  |
| Participant flow diagram (strongly recommended) | [#13a](https://www.goodreports.org/reporting-checklists/consort/info/#13a) | For each group, the numbers of participants who were randomly assigned, received intended treatment, and were analysed for the primary outcome |  | 10 |
| Participant flow | [#13b](https://www.goodreports.org/reporting-checklists/consort/info/#13b) | For each group, losses and exclusions after randomization, together with reason |  | 10 |
| Recruitment | [#14a](https://www.goodreports.org/reporting-checklists/consort/info/#14a) | Dates defining the periods of recruitment and follow-up |  | 4, 5 |
| Recruitment | [#14b](https://www.goodreports.org/reporting-checklists/consort/info/#14b) | Why the trial ended or was stopped |  | N/A |
| Baseline data | [#15](https://www.goodreports.org/reporting-checklists/consort/info/#15) | A table showing baseline demographic and clinical characteristics for each group |  | 10, 11 |
| Numbers analysed | [#16](https://www.goodreports.org/reporting-checklists/consort/info/#16) | For each group, number of participants (denominator) included in each analysis and whether the analysis was by original assigned groups |  | 13, 14 |
| Outcomes and estimation | [#17a](https://www.goodreports.org/reporting-checklists/consort/info/#17a) | For each primary and secondary outcome, results for each group, and the estimated effect size and its precision (such as 95% confidence interval) | For the outcome(s) for which non-inferiority/equivalence was hypothesized, a figure showing confidence intervals and the non-interiority/equivalence margin may be useful | 13, 14/11,13 |
| Outcomes and estimation | [#17b](https://www.goodreports.org/reporting-checklists/consort/info/#17b) | For binary outcomes, presentation of both absolute and relative effect sizes is recommended |  | 13 |
| Ancillary analyses | [#18](https://www.goodreports.org/reporting-checklists/consort/info/#18) | Results of any other analyses performed, including subgroup analyses and adjusted analyses, distinguishing pre-specified from exploratory |  | N/A |
| Harms | [#19](https://www.goodreports.org/reporting-checklists/consort/info/#19) | All important harms or unintended effects in each group (For specific guidance see CONSORT for harms) |  | N/A |
| Conclusions | [#20](https://www.goodreports.org/reporting-checklists/consort/info/#20) | General interpretation of the results | Interpretation taking into account the non-inferiority/equivalence hypotheses and any superiority hypotheses | 18/18 |
| **Discussion** |  |  |  |  |
| Limitations | [#21](https://www.goodreports.org/reporting-checklists/consort/info/#21) | Trial limitations, addressing sources of potential bias, imprecision, and, if relevant, multiplicity of analyses |  | 18 |
| Generalisability | [#22](https://www.goodreports.org/reporting-checklists/consort/info/#22) | Generalisability (external validity, applicability) of the trial findings |  | 16 |
| Interpretation | [#23](https://www.goodreports.org/reporting-checklists/consort/info/#23) | Interpretation consistent with results, balancing benefits and harms, and considering other relevant evidence | Interpret results in relation to the non-inferiority/equivalence hypothesis. lf a superiority conclusion is drawn for outcome(s) for which non-inferiority/equivalence was hypothesized, provide justification for switching | 16, 17/16,17 |
| Registration | #24 | Registration number and name of trial registry |  | 2 |
| **Other information** |  |  |  |  |
| Interpretation | [#25](https://www.goodreports.org/reporting-checklists/consort/info/#25) | Interpretation consistent with results, balancing benefits and harms, and considering other relevant evidence |  | 16,17 |
| Protocol | #26 | Where the full trial protocol can be accessed, if available |  | 4 |
| Funding | #27 | Sources of funding and other support (such as supply of drugs), role of funders |  | 18,19 |

None The CONSORT checklist is distributed under the terms of the Creative Commons Attribution License CC-BY. This checklist can be completed online using <https://www.goodreports.org/>, a tool made by the [EQUATOR Network](https://www.equator-network.org) in collaboration with [Penelope.ai](https://www.penelope.ai)
