# Supplementary material for: Modified combined short and long axis method versus oblique axis method in adult patients undergoing right internal jugular vein cannulation: A randomized controlled non-inferiority study
Source: PLoS One. 2023 Dec 19;18(12):e0295916. doi: 10.1371/journal.pone.0295916 (PMC10729954; doi:10.1371/journal.pone.0295916)
Supplement: Attachment — Submitted filename: Response to Reviewers.docx [file pone.0295916.s006.docx]

Jiaxi Tang MD

First Affiliated Hospital of Chongqing Medical University, Chongqing, China

Chongqing University Cancer Hospital, Chongqing 400030, China

E-mail: tangjiaxi1029@126.com

Sep 25, 2023

**RE:  Manuscript ID:** PONE-D-23-27163

Dear Editor and Reviewers，

We would like to thank the editor for giving us a chance to resubmit the paper, and also thank the reviewers for giving us constructive suggestions which would help us both in English and in depth to improve the quality of the paper. Here we submit a revised version of our manuscript with the title “**Modified combined short and long axis method versus oblique axis method in adult patients undergoing right internal jugular vein cannulation: A randomized controlled non-inferiority study**”, which has been modified according to the reviewers’ suggestions. Efforts were also made to correct the mistakes and improve the English of the manuscript. We track all the changes in the revised manuscript.

Sincerely yours,

Jiaxi Tang MD

-------------------------------------------------------------------------------------------------------

The following is a point-to-point response to editor and the three reviewers’ comments.

**Editor:**

Specific Comments:

**Editor:** When submitting your revision, we need you to address these additional requirements.

Please ensure that your manuscript meets PLOS ONE's style requirements, including those for file naming. The PLOS ONE style templates can be found at

https://journals.plos.org/plosone/s/file?id=wjVg/PLOSOne_formatting_sample_main_body.pdf and

https://journals.plos.org/plosone/s/file?id=ba62/PLOSOne_formatting_sample_title_authors_affiliations.pdf

**Answer:** Thank you for your guidance and assistance in our submission process. Your efficient and responsible work is admirable. We have checked the format of the manuscript again to confirm that our manuscript meets PLOS ONE's style requirements.

**Reviewer #1:**

Specific Comments:

**Reviewer #1:** Thank you for allowing me to review "Modified combined short and long axis method versus oblique axis method in adult patients undergoing right internal jugular vein cannulation: A randomised controlled non-inferiority study" by Jia-Xi Tang et al.

The authors compared first needle pass without posterior vessel wall puncture between modified combined short and long axis method (MCSL) and oblique axis in-plane method (OA-IP). The first needle pass without posterior vessel wall puncture was not significantly different between the two groups.

This is an interesting study. However, I may be mistaken, but there may be a critical problem.

My suggestions are as follows.

American and British English are mixed.

Introduction

“the operator's puncture posture is very awkward”

I have never used the OA-IP method. Please explain how it is awkward.

Methods

Intervention

Only one operator performed all IJVC. This is a very serious limitation.

Study endpoints and sample size calculation ”We estimated that 85 patients per group would supply 90% power at a level of 0.025 to compare the MCSL approach with the OA-IP method.“

Do you mean “at an α level” ?

Furthermore, using the numbers you indicated and doing a sample size calculation, I came up with 1615 per group, even as a one-tailed test. I am no expert in statistics, but I think the journal should check to see if the author’s sample size calculation is correct.

Primary outcome

“The difference in rate (MCSL vs OA-IP) was 4.2% (95% CI: -5.5-13.6), which confirmed the non-inferiority with the margin of -8%.”

Since the 95% confidence interval includes a margin, I don't think non-inferiority has been proven.

Reference11

Watanabe K, Tokumine J, Lefor AK, et al.

**Answer:** Thank you for the comments on the paper. Thanks for your affirmation of this article. Thank you for your pertinent comments, which have greatly helped to improve the quality and rigor of the article. We have made corresponding revisions according to your comments.

1. American and British English are mixed.

**Answer:** Thank you for the comments on the paper, which will greatly help to improve the accuracy of the English use of the manuscript. We have carefully checked the error of the misuse of American and British English and also corrected errors in syntax and text formatting. (Line 3, 27, 29, 34, 36, 57, 59, 63, 76, 84, 87-89, 104, 107, 122, 134, 135, 163, 177, 178, 180, 186, 187, 190, 196, 197, 215, 219, 229, 243, 248, 250, 251, 258, 300, 366, 371, 521, 522)

2. Introduction

“the operator's puncture posture is very awkward”

I have never used the OA-IP method. Please explain how it is awkward.

**Answer:** Thank you for the comments and reminders on the paper, which will increase the reader's understanding of the document. We have added a description of the OA-IP operation details and why this operation is awkward. (Line 64-68)

In addition, we have uploaded two pictures of OA-IP puncture operation to help reviewers understand the OA-IP puncture operation method. (see attachment1)

3. Methods

Intervention

Only one operator performed all IJVC. This is a very serious limitation.

**Answer:** Thank you for your insights and pointing out the shortcomings of our research, which is very enlightening for us. In order to reduce the difference in technical level of operators, we adopted a single-person operation design. This design does have limitations, and we also stated this limitation in the manuscript. We will use more reasonable designs in future studies to reduce the impact of such biases on experimental results. Thanks again to the reviewers for their sincere comments. (Line 116, 117, 351, 352)

4. Study endpoints and sample size calculation ”We estimated that 85 patients per group would supply 90% power at a level of 0.025 to compare the MCSL approach with the OA-IP method.“Do you mean “at an α level” ?

Furthermore, using the numbers you indicated and doing a sample size calculation, I came up with 1615 per group, even as a one-tailed test. I am no expert in statistics, but I think the journal should check to see if the author’s sample size calculation is correct.

**Answer:** Thank you for your comments and questions, which are necessary for scientific rigor. We used PASS software (version 15) to calculate the sample size of the study, using the sample size calculation formula for non-inferiority studies. Please see attachment 2 for the specific calculation process. In addition, we thank you for correcting the problems in our presentation, indeed at the α level. We have corrected the issue. By calculating again, we found that there is a slight difference from the previous calculation. We have modified it based on the latest calculation, and there is no difference in the total sample size. (Line 188, 189)

5. Primary outcome

“The difference in rate (MCSL vs OA-IP) was 4.2% (95% CI: -5.5-13.6), which confirmed the non-inferiority with the margin of -8%.”

Since the 95% confidence interval includes a margin, I don't think non-inferiority has been proven.

Reference11

Watanabe K, Tokumine J, Lefor AK, et al.

**Answer:** Thank you for your comments. We are sorry that our writing may have misled the reviewers. In our study, the mean difference in Primary outcome was 4.2% in MCSL vs OA-IP, and the 95% confidence interval was -5.5% to 13.6%. This confidence interval did not include -8%, so we believe that non-inferiority can be proved. We have standardized the use of hyphens so that hyphens can be distinguished from minus signs (Line 29, 36, 123, 200, 217, 219, 224, 233, 251).

**Reviewer #2:**

Specific Comments:

**Reviewer #2:** Thank you for the opportunity to revise this interesting randomized trial by Tang et al. on the comparison between combined short and long axis method and oblique axis method in adult patients undergoing right internal jugular vein cannulation. The paper is sound and original. However, there are some issues that need to be addressed:

- I believe there is an issue with the reference style, as I don't think they should be in superscript in the manuscript.

- Line 48-49. Authors should also add that ultrasound guided cannulation has proven to be safer and better than landmark technique regardless the cannulation site (doi: 10.1097/CCM.0000000000005819 - doi: 10.1002/14651858.CD01144). Please briefly discuss and cite these 2 references.

- Line 56. Why is the operator's puncture posture very awkward? Please specify.

- The manuscript must be edited for English, as there are some minor mistakes, like "have" instead of "has" in line 73.

- Line 140. Why you did not use triple or quadruple lumen cathethers? Please specify the caliber in French of the catheters used.

- Please increase the text size in all the Tables presented to increase readability.

**Answer:** Thank you for your comments and valuable suggestions, which will greatly help to improve the quality and readability of the manuscript. We have revised it according to the reviewer's comments. Thank you again for your review and hard work.

1. I believe there is an issue with the reference style, as I don't think they should be in superscript in the manuscript.

**Answer:** Yes, your opinions inspired us and we revised the manuscript accordingly. (Line 49, 51, 54, 57, 58, 60, 63, 74, 87, 89, 135, 266, 268, 272, 280, 285, 302)

2. Line 48-49. Authors should also add that ultrasound guided cannulation has proven to be safer and better than landmark technique regardless the cannulation site (doi: 10.1097/CCM.0000000000005819 - doi: 10.1002/14651858.CD01144). Please briefly discuss and cite these 2 references.

**Answer:** Thank you for your references. These two articles are very valuable, which are very important to enrich and clarify the point of view of our manuscript. We have briefly discuss and cite these 2 references. (Line 57, 416-424).

3. Line 56. Why is the operator's puncture posture very awkward? Please specify.

**Answer:** Thank you for the comments and reminders on the paper, which will increase the reader's understanding of the document. We have added a description of the OA-IP operation details and why this operation is awkward (Line 64-68).

In addition, we have uploaded two pictures of OA-IP puncture operation to help reviewers understand the OA-IP puncture operation method. (see attachment1)

4. The manuscript must be edited for English, as there are some minor mistakes, like "have" instead of "has" in line 73.

**Answer:** Thank you for your careful and responsible review and help, which has greatly helped to improve the quality and readability of the manuscript. We have carefully checked for similar errors. Thanks again. (Line 57, 59, 87, 107, 300, 366)

5. Line 140. Why you did not use triple or quadruple lumen cathethers? Please specify the caliber in French of the catheters used.

**Answer:** We are sorry that we did not use triple or quadruple lumen cathethers, since our institution did not purchase these types of catheter.

Thank you for your reminder and guidance. Specifying the size of the catheter makes the writing of the manuscript more standardized, easier for readers to understand, and increases the comparability of different studies. (Line 156, 219)

6. Please increase the text size in all the Tables presented to increase readability.

**Answer:** Thank you for your helpful advice, which are very important to improve the readability of the article. We have increased the font size in all the tables to 12. (Line 104, 219, 233)

**Reviewer #3:**

Specific Comments:

**Reviewer #3:** I am pleased to commend you on your research paper titled "Modified Combined Short and Long Axis Method Versus Oblique Axis Method in Adult Patients Undergoing Right Internal Jugular Vein Cannulation: A Randomized Controlled Non-Inferiority Study." Your study exhibits a robust scientific approach and has made a valuable contribution to the field of medical procedures. The meticulous design of your randomized controlled non-inferiority study reflects a high level of scientific rigor. Your choice of research methodology and the focus on a topic as important as vein cannulation in adult patients demonstrate your dedication to improving medical practices. In conclusion, I believe that your study has the potential to make a positive impact in the field of medical procedures and should be accepted for publication with the suggested revisions. While your research is indeed noteworthy, I would recommend minor revisions and an English language edit to enhance the clarity and readability of the manuscript. These revisions will ensure that your valuable findings can be effectively communicated to a broader audience.

Introduction: The introduction is the weakest part of the manuscript pleas consider English language edit

47 there is a growing body of evidence supporting the use of ultrasound-guided subclavian access: RCTs and meta-analysis. It might be a preferred option for ICU patients. Consider providing information for the reader about new evidence and different characteristics for CVC in OR and ICU population.

57 please restrain from using colloquial words like awkward and use scientific descriptions.

58 it would be more appropriate to use structures rather than organs

Methods:

Sound scientific methodology with study register and CONSORT statement.

patients were randomized appropriately.

Please move sample size calculation to methods section

Intervention:

Detailed description of the intervention.

Study endpoint:

Please define probe placement difficulty, needle visualization

Secondary outcomes:

Would it be possible to differentiate mechanical complications? please provide n for artery puncture, hematoma, pneumothorax etc. It will help to include your research in future meta-analysis

Discussion:

There was a mean difference of 25 s of cannulation time. Is it clinically relevant difference?

The authors use a rigorous definition of first needle pass without PVWP. The success rate is exceptionally high do you think that your results would be replicable with less experienced operators? Do you consider MCSL an advanced US technique? What would be the learning curve ? Interested to hear your opinion

Discussion section could shortened and more focused

**Answer:** Thank you for your review, which makes a huge contribution to the dissemination of science. Thank you very much for your recognition and valuable comments on our paper. We will improve our manuscript based on your comments.

1. Introduction: The introduction is the weakest part of the manuscript pleas consider English language edit

**Answer**: Thank the reviewer for the comments. We have checked the manuscript for language errors and inauthentic expressions, and we have also asked native English speakers to help check the manuscript for language problems, hoping to increase the readability and accuracy of the manuscript. ( Line 48-57, 59, 87).

2. 47 there is a growing body of evidence supporting the use of ultrasound-guided subclavian access: RCTs and meta-analysis. It might be a preferred option for ICU patients. Consider providing information for the reader about new evidence and different characteristics for CVC in OR and ICU population.

**Answer**: Thank you for your constructive comments, which are very important for readers to have a comprehensive understanding of the background knowledge of this research field. We have added new advances related to ultrasound-guided subclavian venipunctures, and different characteristics for CVC in OR and ICU population. (Line 48-53).

3. 57 please restrain from using colloquial words like awkward and use scientific descriptions.

**Answer**: Thank the reviewer for the comments. We have replaced colloquial words with more scientific expressions (Line 66-69, 306, 312).

4. 58 it would be more appropriate to use structures rather than organs

**Answer**: Thank you for your guidance and help, which is very important to improve the quality of the manuscript. We have made changes according to your guidance (Line 70).

5. Methods:

Sound scientific methodology with study register and CONSORT statement.

patients were randomized appropriately.

Please move sample size calculation to methods section

**Answer**: Thank you for your affirmation and comments. This may be because we did not use the number subheading, leading to misunderstanding. According to the requirements of the magazine, we have used font size to distinguish between titles and sub-titles. In our manuscript, the Sample size calculation font is 16, which belongs to the Methods section (Line 82, 157).

6. Intervention:

Detailed description of the intervention.

**Answer**: Thank you for your suggestions and comments. In the intervetion section, we describe the information of the equipment used in this study, the information of the operator, the content of data collection, and the operation steps of the two puncture methods. If there are any gaps in our description, please kindly advise the reviewer. Thank you (Line 115-156).

7. Study endpoint:

Please define probe placement difficulty, needle visualization

**Answer:** Thank you for your comments and valuable suggestions, which are of great help to readers in understanding our research. We have refined the content of this section (Line 161-163, 168, 169).

8. Secondary outcomes:

Would it be possible to differentiate mechanical complications? please provide n for artery puncture, hematoma, pneumothorax etc. It will help to include your research in future meta-analysis

**Answer:** Thank the reviewer for the comments and your suggestion is greatly appreciated. Maybe we haven't stated it clearly, but we have distinguished the different complications and organized these results in Table 3. To prevent ambiguity, we have made some minor changes to the table 3. The total mechanical complications in the table 3 is the sum of all complications (Line 233, see Table 3).

9. Discussion:

There was a mean difference of 25 s of cannulation time. Is it clinically relevant difference?

**Answer:** Thank you for your review and for asking this meaningful question. The average total cannulation time is about 200 seconds. Compared with the OA-IP group, the MCSL group saves about 25 seconds in the operation of each patient, about 1/10 of the time. Overall, it will improve work efficiency and have a positive effect on busy clinical work. Because this indicator is not the main indicator we care about, the clinically meaningful difference that this indicator needs to achieve is not preset.

10. The authors use a rigorous definition of first needle pass without PVWP. The success rate is exceptionally high do you think that your results would be replicable with less experienced operators?

**Answer:** Thank the reviewer for the comments. Our study chose a more stringent metric, and we achieved a fairly high success rate. For this reason, which we mentioned in the Discussion section, our operators were very skilled practitioners and had become proficient in both puncture methods before conducting this study. If it were performed by a novice who was completely unfamiliar with the two methods, or even unfamiliar with ultrasound-guided vascular intervention, perhaps the success rate would not be so high. Fortunately, both ultrasound-guided methods have a flat learning curve and can be mastered quickly. (Line 268-274).

11. Do you consider MCSL an advanced US technique? What would be the learning curve ? Interested to hear your opinion.

**Answer:** Thank you for your review and your interest in this technology and research, we are very willing to share it. First and foremost, we firmly believe that MCSL is an invaluable technique that amalgamates the strengths of both the short axis and the long axis approaches. Furthermore, it leverages ultrasound artifacts for precise puncture site localization, thereby ensuring puncture accuracy. Additionally, the learning curve associated with this method is relatively gentle, making it particularly beginner-friendly.

We were under the impression that mastering this technique would require only a few cases. Unfortunately, we did not collect data regarding the learning process for this technique. In future research, we anticipate well-designed studies to assess the learning curve for novices, with the aim of obtaining accurate learning curve data.

12. Discussion section could shortened and more focused

**Answer:** Thank the reviewer for the comments and your suggestion is greatly appreciated. We have condensed the discussion section by removing redundant portions (Line 264-341).
