# Supplementary material for: Modified combined short and long axis method versus oblique axis method in adult patients undergoing right internal jugular vein cannulation: A randomized controlled non-inferiority study
Source: PLoS One. 2023 Dec 19;18(12):e0295916. doi: 10.1371/journal.pone.0295916 (PMC10729954; doi:10.1371/journal.pone.0295916)
Supplement: Attachment — Submitted filename: Response to Reviewers_R2.docx [file pone.0295916.s007.docx]

Jiaxi Tang MD

First Affiliated Hospital of Chongqing Medical University, Chongqing, China

Chongqing University Cancer Hospital, Chongqing 400030, China

E-mail: tangjiaxi1029@126.com

Nov 22, 2023

**RE:  Manuscript ID:** PONE-D-23-27163R1

Dear Editor and Reviewers，

We would like to thank the editor for giving us a chance to resubmit the paper, and also thank the reviewers for giving us constructive suggestions which would help us both in depth and language to improve the quality of the paper. Here we submit a revised version of our manuscript with the title “**Modified combined short and long axis method versus oblique axis method in adult patients undergoing right internal jugular vein cannulation: A randomized controlled non-inferiority study**”, which has been modified according to the reviewers’ suggestions. We track all the changes in the revised manuscript.

Sincerely yours,

Jiaxi Tang MD

-------------------------------------------------------------------------------------------------------

The following is a point-to-point response to editor and the four reviewers’ comments.

**Editor:**

Specific Comments:

**Editor:** Please include the following items when submitting your revised manuscript:

1. A rebuttal letter that responds to each point raised by the academic editor and reviewer(s). You should upload this letter as a separate file labeled 'Response to Reviewers'.

2. A marked-up copy of your manuscript that highlights changes made to the original version. You should upload this as a separate file labeled 'Revised Manuscript with Track Changes'.

3. An unmarked version of your revised paper without tracked changes. You should upload this as a separate file labeled 'Manuscript'.

4. Please revise according to the comments provided by reviewer 1 and reviewer 4.

**Answer:** Thank you for your guidance and assistance in our submission process. Your efficient and responsible work is admirable. We will revise our manuscript according to the editor and reviewers and submit the revised manuscript.

**Reviewer #1:**

Specific Comments:

**Reviewer #1:** Thank you for allowing me to review "Modified combined short and long axis method versus oblique axis method in adult patients undergoing right internal jugular vein cannulation: A randomised controlled non-inferiority study" by Jia-Xi Tang et al.

The authors addressed the reviewer’s comments. I confirmed that the sample size calculation was correct and that non-inferiority was proved.

I have only one comment.

Limitation

The authors stated that “The operator in this study was an attending physician, and his proficiency in both procedures may influence the results of the trial.” in the limitation section.

However, I think it should be emphasized that there is only one operator.

**Answer:** Thank you for reviewing the manuscript again and for your efforts in improving the quality of our manuscript. We also appreciate your affirmation and your pertinent opinions. We have made corresponding revisions according to your comments.

1. Limitation

The authors stated that “The operator in this study was an attending physician, and his proficiency in both procedures may influence the results of the trial.” in the limitation section.

However, I think it should be emphasized that there is only one operator.

**Answer:** Thank you for the comments on the paper, which are very enlightening and accurately point out the limitation of our research design. We have made corresponding modifications in accordance with your comments to more clearly state the limitations of our research. (Line 299-301)

**Reviewer #2:**

Specific Comments:

**Reviewer #2:** Dear authors, all the comments were successfully addressed. I believe the paper is now ready for acceptance.

**Answer:** Thank you for your previous comments and valuable suggestions, which greatly help to improve the quality and readability of the manuscript. In addition, thank you for your recognition of our work and your praise for our research. Thank you again for your hard work.

**Reviewer #3:**

Specific Comments:

**Reviewer #3:** Thank you for your prompt revisions and thorough attention to the reviewers' comments. I have carefully reviewed the updated manuscript, and I am pleased to confirm that all the suggested changes have been successfully addressed.

I have no further comments at this time, and I appreciate your diligence in improving the manuscript. Your efforts have significantly strengthened the overall quality of the work.

I look forward to seeing the final version of your manuscript.

**Answer:** Thank you for your outstanding efforts and contributions to improving the quality of our manuscript, and for your recognition and appreciation of our work.

**Reviewer #4:**

Specific Comments:

**Reviewer #4:** The authors present the results of a randomised controlled non-inferiority study with a two arm parallel group design to compare MCSL method with OAIP method with repect to first needle pass without posterior vessel wall puncture (PVWP) in patients with IJVC patients. They concluded that MCSL is non-inferior to OA-IP.

In general, the presentation needs revision with respect to clarification of sample size justification, and non-inferiority analysis.

• Please provide a CONSORT checklist for non-inferiority studies (PLoS Med. 2010;7(3):e1000251. PMID: 2035206). (Please address every aspect of the checklist within the paper.)

• (formulate of non-inferiority hypothesis): Please formulate the specific non-inferiority hypothesis. The description does not specify whether it should be proven that MCSL is non-inferior to OA-IP or vice versa in relation to the non-inferiority margin of 8%.

• (analysis of primary endpoint variable (PeV): To proof the non-inferiority hypothesis a one-sided 95% confidence interval should be given. This should be calculated for the ITT as well as PP population to confirm rejection of the null hypothesis. (see abstract and result section)

L28: Please rephrase: ...primary outcome is the event of first .....

L34: see analysis of PeV

L41: As the register is not accessible, please provide a English translated confirmed entry of data.

L70: see formulate of non-inferiority hypothesis

L72: Compare is misleading term here.

L76: Evaluator blinded is not clear. Please explain, who assessed the PeV and how blinding is reached.

L80: Use CONSORT for non-inferiority

L94: PBR(4) results in a high number of predictable allocations, here at least 47 up to 95 predictable allocations, in particular with implementation by sealed opaque envelops. This causes allocation bias and need to be discussed in the limitation section.

L99. Please explain the meaning and implementation process to blinding of monitors and stat analysts with respect to detection, performance and attrition bias.

L144ff: Please describe the time and measurement method of the PeV - PVWP. Here in particular the validity of the measurement methods, i.e. objective measurement methods (measurement free of knowledge about allocated treatment), needs to be described.

L171: I could not follow the sample size calculation. My program results in 186/group using the stated numbers. Please provide details of the input data, statistical rule for calculation of the confidence interval as main analysis and software program used for computation.

L173: Please justify the non-inferiority margin, e.g. by expected difference of standard to placebo.

L177: Please describe the analysis populations.

L186: Rephrase according to analysis of PeV.

L193: Skip statistical tests on baseline data, not meaningful in RCT's.

L196: Please skip data across group = overall data. Not meaningful.

L204: Significance test is not meaningful here. Use the one sided 95% confidence interval for argumentation and show how the CI is similar in ITT and PP.

L273: Avoid reporting of results in the discussion section.

L281: Limitation section should focus on operational and methodological aspects of the study. Include discussion of sample size justification, if needed and allocation bias.

**Answer:** Thank you for your professional and valuable comments and substantial contribution to improving the quality and rigor of our manuscript. We have revised our manuscript based on your comments.

1. Please provide a CONSORT checklist for non-inferiority studies (PLoS Med. 2010;7(3):e1000251. PMID: 2035206). (Please address every aspect of the checklist within the paper.)

**Answer**: Thank the reviewer for the comments. We carefully read the CONSORT checklist for non-inferiority and presented the content required in the checklist into our manuscript, and we provided the CONSORT checklist for non-inferiority in the supporting document (S1 Table). We also cited the corresponding literature in the manuscript.( Line 82-84).

2. (formulate of non-inferiority hypothesis): Please formulate the specific non-inferiority hypothesis. The description does not specify whether it should be proven that MCSL is non-inferior to OA-IP or vice versa in relation to the non-inferiority margin of 8%.

**Answer**: Thank you for your constructive comments, which are very important for readers to have a comprehensive understanding of the background knowledge of this research field and the rationale of our non-inferiority margin choice.

Because the oblique axis in-plane method (OA-IP) is currently a widely recognized central venous puncture method, it combines the advantages of short-axis and long-axis approaches, thereby increasing the success rate of puncture. Our puncture method is a novel approach that also integrates the advantages of both short-axis and long-axis techniques. Moreover, our method strategically selects puncture points away from anatomical structures such as the external jugular vein and brachial plexus nerves, making it theoretically safer. Therefore, we intend to demonstrate through a non-inferiority study that our method is not inferior to OA-IP in terms of puncture success rate.

Typically, the non-inferiority margin for propotion is set at 10-15%. Following discussions within our professional group, we have opted for a more stringent threshold of 8%. In other words, we consider that if the success rate of our Modified Combined Short and Long-axis (MCSL) method is below 8% compared to OA-IP, it cannot be deemed non-inferior. We have added the rationale for selecting the 8% threshold to our manuscript based on your advice. (Line 55-72).

3. (analysis of primary endpoint variable (PeV): To proof the non-inferiority hypothesis a one-sided 95% confidence interval should be given. This should be calculated for the ITT as well as PP population to confirm rejection of the null hypothesis. (see abstract and result section)

**Answer**: Thank the reviewer for the comments. When establishing non-inferiority, we used a two-sided 95% confidence interval (CI), which is equivalent to a one-sided 97.25% CI, which has greater accuracy than a one-sided 95% CI (JAMA. 2012 Dec 26;308(24):2594-604. PMID: 23268518. DOI: 10.1001/jama.2012.87802). In our study, despite three patients not completing the assigned treatment and being categorized as puncture failures (surpassing three puncture attempts (n=2) and arterial puncture (n=1)), it is crucial to note that the non-inferiority indicator focuses on the puncture success rate, and data collection was completed for all patients. Consequently, for the statistical analysis of the non-inferiority indicator, we opted for an Intent-to-Treat (ITT) analysis over a Per-Protocol (PP) analysis.

Appreciating your advice, we conducted a PP analysis in the PeV, revealing a rate difference of 7.1% (MCSL (85/92, 92.4%) vs. OA-IP (81/95, 85.3%)) with a 95% CI of -1.8% to 16.1%. Both ITT and PP analyses affirmed the non-inferior conclusion. (Line 191, 192, 200).

4. L28: Please rephrase: ...primary outcome is the event of first .....

**Answer**: Thank you for your guidance and help, which is very important to increase the accuracy of presentation and improve the quality and readability of the manuscript. We have made changes according to your guidance (Line 28).

5. L34: see analysis of PeV

**Answer**: Thank you for your comments. After your reminder, we noticed the inaccuracy in our statement and we have revised the error. Thank you again for your rigorous and careful review, which is important to maintaining academic rigor and the readability of the manuscript. (Line 34).

6. L41: As the register is not accessible, please provide a English translated confirmed entry of data.

**Answer**: Thank you for your suggestions and comments. Line L41 is the project registration information we submitted, including the registration website (Chinese Clinical Trial Registry (ChiCTR)) and registration number (ChiCTR2100046899). We have provided the website address (https://www.chictr.org.cn/) in the method section. Please enter our registration information according to the steps we provide to enter the registration URL (see attachment 3) (Line 80).

In addition, we have also shared our metadata on the DRYAD public data platform, and we have provided the URL to enter and download the our metadata (see Data Review URL).

7. L70: see formulate of non-inferiority hypothesis

**Answer:** Thank you for your commens and reminder. We realize that our previous manuscript did not state the details of the hypothesis clearly. We have already stated the details of the hypothesis (Line 71,72).

8. L72: Compare is misleading term here.

**Answer:** Thank the reviewer for the comments and your suggestion is greatly appreciated. We have replaced compare with more accurate words. Thank you for your professional guidance on the language of our manuscript, which is very important to increase the readability of the manuscript (Line 73).

9. L76: Evaluator blinded is not clear. Please explain, who assessed the PeV and how blinding is reached.

**Answer:** Thank you for your review and for asking this meaningful question. We apologize for our negligence in preparing the manuscript and for not attracting readers' attention in particular. We have listed the outcome indicator evaluation in a separate paragraph. We hope that our statement is clear enough. Thank you again for your valuable opinions (Line 177-182).

10. L80: Use CONSORT for non-inferiority

**Answer:** Thank the reviewer for the comments. We have added a non-inferiority CONSORT statement, reported our research based on the non-inferiority CONSORT statement, and cited the corresponding literature. (Line 82-84, see S1 Table).

11. L94: PBR(4) results in a high number of predictable allocations, here at least 47 up to 95 predictable allocations, in particular with implementation by sealed opaque envelops. This causes allocation bias and need to be discussed in the limitation section.

**Answer:** Thank you for your professional review and valuable advice. Indeed, if the operator knows that the randomization method is block randomization and the block is 4, he can guess the allocation plan. Therefore, when we generate the randomization plan, it is completed by statistical experts, and the randomization method blinds both the operator and the outcome indicator evaluator. And our random allocation plan is also kept by statistical experts. Unblinding was performed only after the experimental and statistical analyzes were completed.

We regret that our previous manuscript did not clearly explain the specific details of randomization generation and allocation concealment. We have added corresponding descriptions, hoping to reduce misleading to readers (Line 101-109).

12. L99. Please explain the meaning and implementation process to blinding of monitors and stat analysts with respect to detection, performance and attrition bias.

**Answer:** Thank the reviewer for the comments. To prevent selection bias caused by the data cleaning and analysis process, we blinded data monitoring and data analysis.

Before the data analysis was completed, only the enrollment numbers of subjects were recorded, and the group names were replaced with A and B. We have added the details about blind execution in the manuscript. (Line 107-109).

13. L144ff: Please describe the time and measurement method of the PeV - PVWP. Here in particular the validity of the measurement methods, i.e. objective measurement methods (measurement free of knowledge about allocated treatment), needs to be described.

**Answer:** Thank you very much for your careful review and professional suggestions. Accurately describing the experimental details can help readers understand the research, and also help subsequent studies to replicate our research. We have added the judgment criteria and methods of the Pev-PVWP, and cited relevant references. As described previously, we performed judgments on ultrasound videotapes by two raters who were not involved in the procedure. (Line 161, 162, 177-182).

14. L171: I could not follow the sample size calculation. My program results in 186/group using the stated numbers. Please provide details of the input data, statistical rule for calculation of the confidence interval as main analysis and software program used for computation.

**Answer:** Thank you for your comments and questions, which are necessary for scientific rigor. We used PASS software (version 15) to calculate the sample size of the study, using the sample size calculation formula for non-inferiority studies. Please see attachment 2 for the specific calculation process. We have added sample calculation software information in the Methods section (Line 186, 187).

15. L173: Please justify the non-inferiority margin, e.g. by expected difference of standard to placebo.

**Answer:** Thank the reviewer for the comments. Our study diverges from a typical drug study as it doesn't involve a placebo. Certainly, we considered the landmark-guided puncture method as a placebo. Unfortunately, through literature review, our comprehensive review did not identify comparative studies on the success rates of the oblique-axis in-plane puncture (OA-IP) method and the landmark-guided jugular vein puncture method. Additionally, our Modified combined short and long axis (MCSL) puncture method is novel, lacking data for comparison with the landmark-guided internal jugular vein puncture method. Based on data from our unpublished preliminary experiments, both methods (OA-IP and MCSL) exhibited a puncture success rate of approximately 90%. Considering common practices in proportion-based research, the non-inferiority margin is typically set at 10-15%. Following group discussions, we opted for a relatively stringent 8%. We have explained in the manuscript the reasons why we chose 8% as the non-inferior margin (Line 71,72).

16. L177: Please describe the analysis populations.

**Answer:** Thank the reviewer for the comments. I'm very sorry. There may be discrepancies in page numbers between the manuscript you reviewed and mine. Line 177 is an explanation of statistical analysis in the methods section. We have described the analysis populations in the methods and results section. Moreover, we have presented the population baseline data of the study in Table 2. If there is anything unclear in the presentation, we hope to get guidance from the reviewers. Thank you again. (Line 86-94, 216).

17. L186: Rephrase according to analysis of PeV.

**Answer:** Thank the reviewer for the comments and your suggestion is greatly appreciated. We have revised the manuscript according to your advice(Line 221-223).

18. L193: Skip statistical tests on baseline data, not meaningful in RCT's.

**Answer:** Thank you for your advice and expertise. Indeed, in large randomized controlled studies, baseline levels are balanced between groups, so we did not perform statistical analysis. However, we present the baseline data of both groups to facilitate data extraction by future researchers conducting systematic reviews.

19. L196: Please skip data across group = overall data. Not meaningful.

**Answer:** Thank the reviewer for the comments and suggestion. thanks for your advice. We've removed lengthy narrative (Line 211-213).

20. L204: Significance test is not meaningful here. Use the one sided 95% confidence interval for argumentation and show how the CI is similar in ITT and PP.

**Answer:** Thank the reviewer for the comments. When establishing non-inferiority, a one-sided 95% confidence interval is equivalent to a two-sided 90% confidence interval, and a one-sided 97.25% CI is equivalent to a two-sided 95% confidence interval (CI). This study used a 2-sided 95% confidence interval to determine non-inferiority, which has greater accuracy than a two-sided 90% CI (JAMA. 2012 Dec 26;308(24):2594-604. PMID: 23268518. DOI: 10.1001/jama.2012.87802). In our study, despite three patients not completing the assigned treatment and being categorized as puncture failures (surpassing three puncture attempts (n=2) and arterial puncture (n=1)), it was crucial to note that the non-inferiority indicator focuses on the puncture success rate, and data collection was completed for all patients. Consequently, for the statistical analysis of the non-inferiority indicator, we opted for an Intent-to-Treat (ITT) analysis over a Per-Protocol (PP) analysis

Appreciating your advice, we conducted a PP analysis, revealing a rate difference of 7.1% (MCSL (85/92, 92.4%) vs. OA-IP (81/95, 85.3%)) with a 95% CI of -1.8% to 16.1%. Both ITT and PP analyses affirmed the non-inferior conclusion (Line 191, 192, 200, 245, 246).

21. L273: Avoid reporting of results in the discussion section.

**Answer:** Thank the reviewer for the comments. We strongly agree with your point that the results should not be reported in the discussion section. So, you don't even see a number in our discussion section.

The description of the regression analysis in the discussion is not a report of the research results; rather, it is our discussion and analysis of these results. Thank you again for your careful review and we greatly admire your hard work. (Line 291-298).

22. L281: Limitation section should focus on operational and methodological aspects of the study. Include discussion of sample size justification, if needed and allocation bias.

**Answer:** Thank the reviewer for the comments. We have updated the limitations of our study to state that our study was conducted by only one physician, which is indeed a bias. In the future, we will consider this issue and design a more reasonable study plan. Regarding the issues of sample size and allocation bias, please see the previous answers section for details. If the reviewer has any questions or suggestions, please contact me and give your valuable suggestions so that we can jointly improve the quality of the manuscript. Thank you again. (Line 299-301).
